# The Impact of Hypothyroidism on Cardiovascular-Related Healthcare Utilization in the US Population With Diabetes

**DOI:** 10.1210/jendso/bvae204

**Published:** 2024-11-18

**Authors:** Marcelo Ramirez, Antonio C Bianco, Matthew D Ettleson

**Affiliations:** Section of Endocrinology, Diabetes, and Metabolism, University of Chicago, Chicago, IL 60637, USA; Section of Endocrinology, Diabetes, and Metabolism, University of Chicago, Chicago, IL 60637, USA; Section of Endocrinology, Diabetes, and Metabolism, University of Chicago, Chicago, IL 60637, USA

**Keywords:** diabetes, hypothyroidism, cardiovascular disease, healthcare expenditure

## Abstract

**Context:**

Suboptimal treatment of hypothyroidism (HT) is associated with adverse cardiovascular disease (CVD) outcomes, for which patients with diabetes mellitus (DM) are at increased risk.

**Objective:**

This study aimed to compare CVD-related healthcare utilization in DM patients with and without HT in the US population.

**Methods:**

Participant data were collected from the Medical Expenditure Panel Survey (MEPS) over 10 years (2011-2020). Medical conditions were identified by ICD-9/ICD-10 codes associated with expenditures. Healthcare utilization outcomes included number of emergency, hospital, and outpatient visits associated with coronary artery disease (CAD), stroke/transient ischemic attack (TIA), or heart failure; prescriptions related to CVD; and number of visits to specialty providers. A propensity score-based fine stratification matching approach was used to balance sociodemographic covariates to determine the relative risk (RR) contributed by HT on CVD-related care utilization.

**Results:**

A total of 15 580 adult participants with DM were identified, of whom 11.9% had treated HT. In the weighted analysis, a significantly greater proportion of participants with HT had CAD and stroke/TIA-associated visits compared to those without HT (respectively, 22.4% vs 17.8%, *P = *.002; and 7.3% vs 5.4%, *P = *.020). In the matched analysis, participants with HT were more likely to see a specialist (cardiology, endocrinology, and nephrology). Participants with HT were more likely to be treated with cholesterol-lowering medications, beta-blockers, and diuretics.

**Conclusion:**

HT as a comorbidity with DM was associated with increased healthcare utilization related to CVD, specifically visits associated with stroke/TIA, increased use of specialty care, and greater utilization of CVD-related medications.

Hypothyroidism (HT) is defined by low thyroid hormone levels and affects around 30 million adults in the United States [1]. Its association with diabetes mellitus (DM) has been widely reported for decades [2]. Multiple studies have shown that thyroid dysfunction is more frequently seen in patients with type 2 DM than in the general population [3], with a prevalence that ranges from 10% to 25% in some study populations [3-5].

The presence of DM has been well-established as a major risk factor for cardiovascular disease (CVD) [6, 7]. The estimated global prevalence of CVD in patients with DM is up to 32% [6] and represents a major cause of morbidity and mortality in this cohort [6]. This translates into increased CVD-related healthcare utilization and costs in patients with DM when compared to the general population [8]. Thyroid dysfunction has also been strongly associated with CVD [7]. The prevalence of coronary artery disease (CAD) in patients with overt and subclinical hypothyroidism is considerably elevated compared to the general population [9, 10]. Additionally, the prescription and use of statins are notably more common in this group [11, 12]. When poorly controlled with levothyroxine, HT has also been associated with certain cardiovascular events, including atrial fibrillation, stroke, heart failure, and overall cardiovascular mortality [13, 14]. While treatment of HT appears to stabilize CVD risk [13], inadequate thyroid hormone control with levothyroxine is common among these patients [15]. Interestingly, an increased prevalence of DM has been associated with low and elevated thyroid-stimulating hormone (TSH) levels in older patients receiving thyroid hormone replacement therapy [16]. This could indicate that adequate thyroid hormone replacement is more difficult to achieve in patients with DM.

Although both DM and HT—both risk factors for CVD—coexist in many patients, the combined impact of both diseases on cardiovascular health outcomes remains uncertain. Due to DM being a potential risk factor for suboptimal HT treatment, we hypothesize that DM patients with HT have a higher risk of CVD than those without HT, and, as a result, have higher CVD-related healthcare utilization. However, we do not yet know if suboptimal HT treatment occurs to such an extent as to lead to a significant increase of CVD-related care on a population level. In this study, we aimed to estimate the utilization of healthcare visits and medications related to CVD among US adults with DM and treated HT. Our goal was to measure the effect of treated HT on the likelihood of utilizing CVD-related care in US adults with DM.

## Research Design and Methods

### Study Design and Population

This is a multi-year cross-sectional study of participants from the Medical Expenditure Panel Survey (MEPS) database from the years 2011-2020. MEPS is a continuous survey of a nationally representative sample of the noninstitutionalized US population, in which medical and payment data are collected from households, medical providers, and employers. The complex panel survey design includes participant weighting to estimate national healthcare expenditure trends. Many of the recorded prescriptions and healthcare visits (including hospitalizations, emergency care events, and outpatient visits) are linked to medical conditions classified by International Classification of Disease (ICD) code through linkage files available from the MEPS website (https://meps.ahrq.gov/mepsweb/). As this study involved only publicly available data, it was deemed exempt from review by an institutional review board.

All participants in the MEPS database from 2011-2020 who were 18 years of age and older at the time of survey were screened for study eligibility. All participants with a diagnosis of DM were included in the study. Diagnoses were determined using the medical conditions file of the MEPS database, which includes reported diagnoses in the household survey. MEPS uses a simplified ICD coding scheme that collapses similar ICD-9 or ICD-10 codes (depending on the survey year) for the most frequent diagnoses. In the ICD-9 coding scheme (2011-2015), type 1 diabetes and type 2 diabetes were combined as a general DM classification. For those with the ICD-10 scheme, only diagnoses of type 2 DM were included (MEPS ICD codes: type 1 DM and gestational DM were excluded). MEPS participants ≥ 18 years of age with DM as a reported medical comorbidity were study eligible. To be classified as having treated HT, the participant had to meet 2 criteria: (i) a diagnosis consistent with HT or other thyroid disease, and (ii) an active prescription for levothyroxine. Participants who only met 1 of the criteria were excluded. Because the use of levothyroxine was included as part of classification of the study groups, participants without available prescription data were excluded.

### Cardiovascular Disease-Related Healthcare Utilization

The primary study outcome was utilization of CVD-related healthcare. This was defined as any healthcare visit (hospitalization, emergency, or outpatient) associated with a MEPS diagnosis code for CAD, stroke/transient ischemic attack (TIA), or heart failure. Secondary outcomes included the number of healthcare visits with specialty providers (cardiology, endocrinology, and nephrology) and the use of CVD- and DM-related medications (sodium-glucose cotransporter type 2 [SGLT-2] inhibitors, glucagon-like peptide 1 [GLP-1] agonists, angiotensin-converting enzyme [ACE] inhibitors, angiotensin receptor antagonists, diuretics, antiplatelet medications, beta-blockers, and cholesterol-lowering medications).

### Sociodemographic Covariates and Comorbidities

Sociodemographic characteristics were collected on all participants. These included age, sex, family income, race (including White, Black, American Indian/Alaskan, Asian/Hawaiian/Pacific Islander, and multiple races), insurance coverage (private, public, or uninsured), and smoking status (smoker, nonsmoker, unknown). Data on the presence of additional comorbidities—hypertension (HTN), hyperlipidemia (HL), and renal disease—were also collected because of their associated risk with CVD. Unlike HTN and HL, chronic kidney disease is not a priority condition in MEPS; thus, reporting of renal disease is more sporadic. We broadened the definition to renal disease (including chronic, acute, and other renal disease) to maximize the capture of renal disease in the MEPS cohort.

### Statistical Analysis

We sought to measure the effect of treated HT on CVD-related care utilization both as an estimate of the national population with DM and after balancing covariates between those with and without HT. This approach was similar to a previous study examining care utilization in participants using different thyroid hormone preparations [17]. Briefly, a weighted bivariate analysis was conducted between the 2 study groups using complex survey analysis (“survey” R package). In the MEPS dataset, each study participant was assigned a sample weight (PERWT**F) to approximate the total number of individuals with DM in the United States for a given survey year. Because the analysis spanned multiple years, sample weights were averaged across the study period (2011-2020) using the pooled linkage file per MEPS analytical guidelines [18]. Due to the potential skewness some variables in MEPS [19], normality for the continuous variable (age) was assessed via the Kolmogorov-Smirnov test. The nonparametric weighted Kruskal-Wallis (age) and Chi-square tests (categorical variables) were used to compare the study groups. Because of the known sex differences in CVD risk, a sex-stratified weighted analysis was performed to assess the individual impact of HT on CVD-related care utilization in the male and female populations.

**Figure 1.**
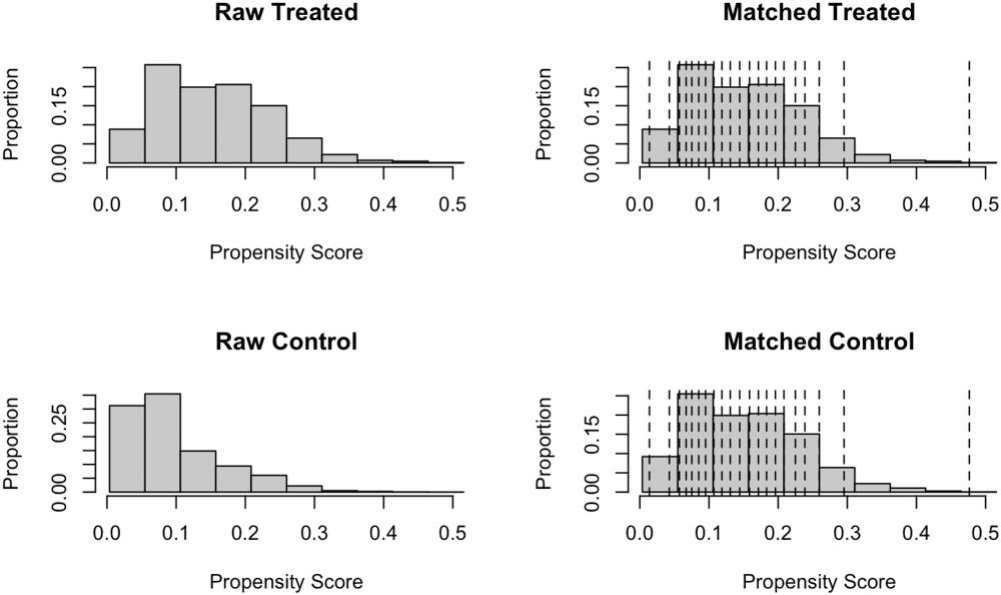
This figure illustrates the distribution of propensity scores for both study groups, before (raw) and after (matched) covariate balancing. The “treated” group includes individuals with hypothyroidism, while the “control” group consists of those without hypothyroidism.

In the second component of the analysis, sociodemographic and clinical covariates were balanced between those with and without HT using a propensity score-based fine stratification approach (“MatchIt” R package). As a result, a control group was formed without HT that was matched for each of the sociodemographic covariates and comorbidities (eg, age, sex, race, insurance coverage, smoking status, HTN, HL, and renal disease) and survey year to the HT group (Fig. 1). A bivariate comparison of CVD-related outcomes was then done similar to the weighted analysis. After covariate balancing, we also estimated the marginal effect (as relative risk) of treated HT on each CVD-related outcome with adjustment for age, sex, race, insurance coverage, family income, smoking status, survey year, and comorbidities. Due to the number of measured outcomes, we applied the Benjamini-Hochberg correction to reduce the false discovery rate (we report both adjusted and unadjusted *P* values). Additionally, in the same fashion, we analyzed the adult cohort without DM (with and without HT) to evaluate the impact of HT on CVD-related care utilization in this population. This provided an opportunity to assess whether any impacts seen in the initial analysis were unique to the DM. This was done using the covariate balanced approach to account for the significant age difference between those with and without HT in the non-DM cohort. All data collection, processing, and statistical testing were completed using R statistical software (version 4.1.2).

## Results

### Study Population

**Figure 2.**
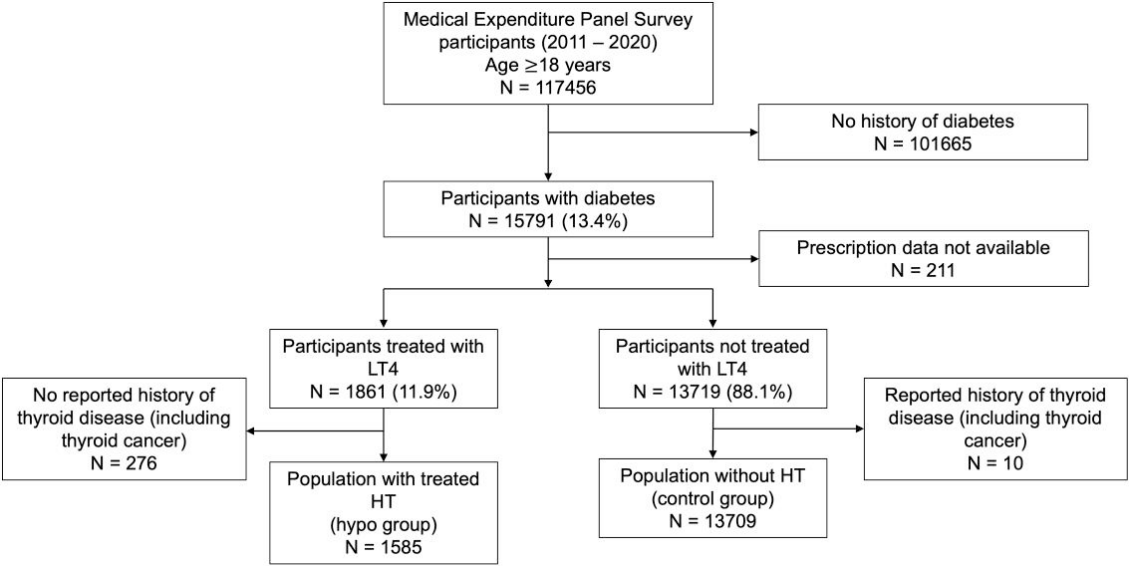
Flow chart of participant selection from the MEPS database. Abbreviations: HT, hypothyroidism; LT4, levothyroxine; MEPS, Medical Expenditure Panel Survey.

A total of 15 580 adults with DM were stratified based on HT diagnosis. Of these, 1585 participants (11.9%) with a history of treated thyroid disease were classified in the treated HT group, while the remaining 13 709 individuals (88.1%) were assigned to the control group (Fig. 2). The 2 study populations differed on several key sociodemographic characteristics, including age, sex, and race/ethnicity. The HT group had a median age of 66 years, while their counterparts without HT had a median age of 62 years (*P* < .001). The HT group had a higher proportion of women (68.9% vs 46.6%, *P* < .001). The majority of subjects with HT and without HT were White, 85% and 74%, respectively (*P* < .001). Insurance coverage was similar between the 2 groups, with 97% coverage in the HT group compared to 95% in those without HT. The family income was lower in the HT group (Table 1). Finally, the prevalence of HTN, HL, and renal disease were higher in the HT group.

**Table 1.** Sociodemographic characteristics of adults with DM stratified by HT (weighted)

	With HT	Without HT	*P* value
Characteristics	N = 1663714	N = 12435990	
Age, median years (IQR)	66.0 (57, 75)	62.0 (52, 71)	<.001
Female sex (%)	1146082.2 (68.9)	5789948.7 (46.6)	<.001
Family income (%)			.013
$0-20000	430373.1 (25.9)	2732390.6 (22.0)	
$20000-50000	506328.3 (30.4)	3859635.2 (31.0)	
$50000-100000	458954.0 (27.6)	3475632.1 (27.9)	
>$100000	268059.1 (16.1)	2368332.3 (19.0)	
Race (%)			<.001
White	1419924.4 (85.3)	9207004.1 (74.0)	
Black	127476.5 (7.7)	2060622.9 (16.6)	
American Indian/Alaskan	18076.2 (1.1)	119649.9 (1.0)	
Asian/Hawaiian/Pacific Islander	61647.1 (3.7)	740086.8 (6.0)	
Multiple races	36590.2 (2.2)	308626.6 (2.5)	
Insurance coverage (%)			<.001
Private	967416.3 (58.1)	7039776.3 (56.6)	
Public	654639.2 (39.3)	4697090.9 (37.8)	
No insurance	41659.0 (2.5)	699123.0 (5.6)	
Smoking status (%)			.006
Smoker	181094.4 (10.9)	1663410.2 (13.4)	
Nonsmoker	1419261.1 (85.3)	10081487.4 (81.1)	
Unknown	63359.0 (3.8)	691092.6 (5.6)	
Comorbidities			
Hypertension	1258731.8 (75.7)	8651328.8 (69.6)	<.001
Hyperlipidemia	1190401.8 (71.6)	7470505.9 (60.1)	<.001
Renal disease	127637.1 (7.7)	479972.8 (3.9)	<.001

Figures are presented as estimates of the national population using individual participant weighting. Abbreviations: DM, diabetes mellitus; HT, hypothyroidism; IQR, interquartile range.

### Weighted Estimates of Utilization of CVD-Related Healthcare

In the weighted bivariate analysis, patients with HT had more visits associated with CAD (22.4% vs 17.8%, adjusted [adj] *P* = .002) and stroke/TIA (7.3% vs 5.4%, adj *P* = .020) than participants without HT. Patients with HT had more healthcare visits with cardiologists (23.3% vs 17.2%, adj *P* = .001), endocrinologists (19.7% vs 11%, adj *P* = .001), and nephrologists (8.5% vs 4.8%, adj *P* = .001) in comparison to their counterparts without HT. Of note, the proportion of participants with at least one visit to a general practice clinician was not significantly different (48.4% vs 46.3%, adj *P* = .279). More patients with HT received at least one CVD-related prescription, including angiotensin receptor blockers (ARBs) (18.6% vs 14.7%, adj *P* = .002), anti-platelets (9.7% vs 7.6%, adj *P* = .038), and cholesterol-lowering medications (70.9% vs 58.5%, adj *P* = .001) (Table 2). In the sex-stratified analysis (Supplementary Table S1) [20], a similar pattern of increased utilization among those with HT was seen in both groups, albeit men had higher utilization in general.

**Table 2.** CVD-related healthcare utilization by adults with DM stratified by HT (weighted)

	With HT	Without HT	*P* value	Adj *P* value
Utilization outcomes	N = 1663714	N = 12435990		
≥ 1 visit associated with primary diagnosis (%)				
CAD	22.4	17.8	.001	.002
Stroke/TIA	7.3	5.4	.012	.020
Heart failure	3.4	2.7	.267	.308
≥ 1 visit with specialty provider (%)				
Cardiology	23.3	17.2	<.001	.001
Endocrinology	19.7	11.0	<.001	.001
Nephrology	8.5	4.8	<.001	.001
General practice	48.4	46.3	.223	.279
CVD-related medications (%)				
SGLT-2 inhibitor	5.2	3.5	.044	.060
GLP-1 agonist	6.0	5.4	.499	.535
ACE inhibitor	34.4	35.1	.672	.672
ARB	18.6	14.7	.001	.002
Diuretic	28.5	22.3	<.001	.001
Antiplatelet	9.7	7.6	.025	.038
Beta-blocker	33.9	28.6	.001	.002
Anti-hyperlipidemia	70.9	58.5	<.001	.001

*P* values were adjusted to reduce false discovery rate using the Benjamini-Hochberg correction. Abbreviations: ACE, angiotensin-converting enzyme; ARB, angiotensin receptor blockers; CAD, coronary artery disease; CVD, cardiovascular disease; DM, diabetes mellitus; GLP-1, glucagon-like peptide 1; HT, hypothyroidism; SGLT-2, sodium-glucose cotransporter 2; TIA, transient ischemic attack.

### Comparison of CVD-Related Healthcare Utilization After Covariate Balancing

In the unweighted analysis with covariate balancing, matched results were similar to those of the weighted analysis in some areas, but the overall impact of HT on care utilization was attenuated. Those in the HT group did not have significantly higher utilization of care related to CAD, stroke/TIA, or heart failure (after *P* value correction was applied). However, similar to the weighted analysis, the HT group was more likely to have at least one visit with cardiology, endocrinology, and nephrology specialists. Other CVD-related medications, including SGLT-2 inhibitors (4.4% vs 3.2%, adj *P* = .030), diuretics (28.8% vs 25.4, adj *P* = .015), beta-blockers (34.8% vs 31.6%, adj *P* = .030), and cholesterol-lowering medications (70.2% vs 65.1%, *P* = .001) were prescribed more in the HT group. Antiplatelet and angiotensin receptor blocker use was no longer associated with HT after covariate balancing (Table 3).

**Table 3.** CVD-related healthcare utilization by adults with DM stratified by HT (matched)

	With HT	Without HT	*P* value	Adj *P* value
Utilization outcomes	ESS = 1585	ESS = 13709		
≥ 1 visit associated with primary diagnosis (%)				
CAD	21.8	19.5	.035	.065
Stroke/TIA	7.5	6.4	.110	.165
Heart failure	3.5	3.2	.470	.588
≥ 1 visit with specialty provider (%)				
Cardiology	22.6	19.2	.002	.010
Endocrinology	18.8	10.0	<.001	.001
Nephrology	8.7	6.6	.005	.015
General practice	49.1	49.5	.740	.740
CVD-related medications (%)				
SGLT-2 inhibitor	4.4	3.2	.014	.030
GLP-1 agonist	5.6	5.3	.673	.721
ACE inhibitor	34.2	36.0	.170	.232
ARB	19.2	17.4	.089	.148
Diuretic	28.8	25.4	.004	.015
Antiplatelet	9.1	8.7	.583	.673
Beta-blocker	34.8	31.6	.014	.030
Anti-hyperlipidemia	70.2	65.1	<.001	.001

*P* values were adjusted to reduce false discovery rate using the Benjamini-Hochberg correction. Balanced covariates: age, sex, race, insurance coverage, family income category, comorbidities, and survey year. Abbreviations: ACE, angiotensin-converting enzyme; ARB, angiotensin receptor blockers; CAD, coronary artery disease; CVD, cardiovascular disease; DM, diabetes mellitus; ESS, estimated sample size; GLP-1, glucagon-like peptide 1; HT, hypothyroidism; SGLT-2, sodium-glucose cotransporter 2; TIA, transient ischemic attack.

Multivariable analysis with the balanced study groups was performed, allowing for adjustment for sociodemographic and clinical covariates and estimation of the marginal effect of HT on utilization outcomes. Notably, adults with HT had a 33% higher risk of requiring a visit for stroke/TIA (RR: 1.33, 95% CI [1.07-1.66], adj *P* = .025) (Table 4). Patients in the HT group were significantly more likely to receive specialized care, with higher rates of consultations with cardiologists (RR: 1.16, 95% CI [1.03-1.31], adj *P* = .026), endocrinologists (RR: 2.01, 95% CI [1.75-2.31], adj *P* = .001), and nephrologists (RR: 1.42, 95% CI [1.15-1.74], adj *P* = .001). Additionally, they were more likely to be prescribed CVD-related medications, including diuretics (RR: 1.14, 95% CI [1.03-1.26], adj *P* = .025), and cholesterol-lowering medications (RR: 1.08, 95% CI [1.04-1.13], adj *P* = .001) compared to patients without HT.

**Table 4.** Multivariable marginal effect relative risk estimates of HT on CVD-related care utilization

Utilization outcomes	RR	95% CI	*P* value	Adj *P* value
≥ 1 visit associated with primary diagnosis (%)				
CAD	1.09	0.96-1.22	.173	.260
Stroke/TIA	1.33	1.07-1.66	.010	.025
Heart failure	1.17	0.87-1.59	.292	.398
≥ 1 visit with specialty provider (%)				
Cardiology	1.16	1.03-1.31	.014	.026
Endocrinology	2.01	1.75-2.31	<.001	.001
Nephrology	1.42	1.15-1.74	<.001	.001
General practice	0.99	0.93-1.07	.977	.977
CVD-related medications (%)				
SGLT-2 inhibitor	1.36	1.11-1.67	.003	.011
GLP-1 agonist	1.12	0.90-1.39	.325	.406
ACE inhibitor	0.97	0.89-1.06	.522	.559
ARB	1.13	0.99-1.29	.070	.117
Diuretic	1.14	1.03-1.26	.009	.025
Antiplatelet	1.09	0.91-1.30	.356	.411
Beta-blocker	1.12	1.02-1.22	.012	.026
Anti-hyperlipidemia	1.08	1.04-1.13	<.001	.001

*P* values were adjusted to reduce false discovery rate using the Benjamini-Hochberg correction. Balanced covariates: age, sex, race, insurance coverage, family income category, comorbidities, and survey year. Abbreviations: ACE, angiotensin-converting enzyme; ARB, angiotensin receptor blockers; CAD, coronary artery disease; CVD, cardiovascular disease; GLP-1, glucagon-like peptide 1; HT, hypothyroidism; RR, relative risk; SGLT-2, sodium-glucose cotransporter 2; TIA, transient ischemic attack.

Results from the covariate balanced analysis in the cohort without DM are shown in Supplementary Tables S2 and S3 [20]. The absolute amount of CVD-related healthcare utilization in all categories was lower in the non-DM cohort, as expected. Having HT appeared to be associated with higher CVD-related care utilization in a similar pattern to the DM cohort, including increased specialty care and use of beta-blockers and cholesterol-lowering medications. However, the effect sizes were generally smaller compared to the DM analysis. The only exceptions were care utilization related to CAD and heart failure, which were higher in the HT group (RR: 1.14, 95% CI [1.02-1.26], adj *P* = .028; RR: 1.41, 95% CI [1.12-1.77], adj *P* = .007, respectively).

## Discussion

In this study, we examined the impact of treated HT on CVD-related healthcare utilization in the US population with DM. Although the relationship between HT and DM has been well-established and the co-incidence of these 2 chronic endocrine disorders is high, the implications of HT on healthcare expenditure outside of thyroid hormone prescriptions have seldom been investigated. Here, after balancing sociodemographic and clinical covariates, we found that patients with DM and treated for HT exhibited a higher frequency of healthcare visits associated with stroke/TIA, more visits with specialty care providers, and received a greater number of prescriptions for CVD-related medications (SGLT-2 inhibitors, diuretics, beta-blockers, and cholesterol-lowering medications). Results were largely similar in the sex-stratified analysis, although the absolute use of CVD-related care was higher in the male cohort.

Because of the high prevalence of DM and HT in the adult US population, the difference in healthcare utilization between the 2 groups represent a significant burden both on patients and the healthcare system. For example, we identified a 4.6% excess of CAD-related care utilization in those with HT. Given our estimate of ∼1.66 million individuals with DM and HT in the weighted analysis, this would represent an estimated excess of 76 500 patients seeking CAD-related care. There are several potential explanations for the increased utilization of healthcare services related to CVD in these patients. Both HT and DM are recognized risk factors for CVD [6, 7], and while effectively managed HT mitigates CVD risk [13], patients with treated HT are often under- or overtreated [15], leading to higher rates of cardiovascular events [13] and worse hospital outcomes [21]. This, in turn, may contribute to a higher usage of healthcare services related to CVD. We also found higher rates of HTN, HL, and renal disease in the HT cohort, which could at least partly explain the increased use of CVD-related care. However, after matching on these comorbidities, several differences persisted, including use of cholesterol-lowering medications and visits to a nephrologist. These results suggest that co-incidence of other CVD risk factors (eg, HTN and HL) likely does not completely explain the observed differences but is a contributing factor.

In the examination of the cohort without DM, we found increased utilization of specialty care and medications in the HT group that mirrored those with DM, but the effect sizes were more modest. These finding suggest that the impact of HT on CVD may be more pronounced in the setting of DM. There are several potential explanations for this. First, it is notable that DM has been associated with increased risk of over- and undertreatment with levothyroxine in older patients [16]. Additionally, poor glycemic control in the setting of DM has been linked with a lower response of thyroid-stimulating hormone to thyrotropin-releasing hormone (TRH) and decreased levels of T3 [22, 23]. Furthermore, HT is known to induce an insulin-resistant state [23-25], which potentially contributes to the escalation of insulin resistance-associated disorders, including CVD. HT has also been associated with an elevated risk of microvascular complications, such as diabetic nephropathy, peripheral neuropathy, and retinopathy among individuals with DM [26-29]. This evidence provides a physiological rationale for why CVD care utilization may be higher in patients with HT and DM vs DM alone.

An alternative explanation is that patients who seek more specialty care may be more likely to be tested and treated for milder forms of HT, for which the implications on CVD risk are less clear [30]. Furthermore, after covariate balancing, our findings that CAD and HF-related care are not increased in the HT group with DM (but are in the DM cohort) suggests that the CVD risk attributable to DM outweighs that of HT, making differences in CVD risk more difficult to detect in the DM cohort. Ultimately, we suspect that both the biological interactions impacting disease control of DM and HT and differences in patient behavior are likely mediators of our observations.

Our study is not exempt from limitations. Given the cross-sectional nature of the study design, we cannot establish a cause-effect relationship between the presence of HT and utilization of CVD-related healthcare in patients with DM. As with many observational studies, there are inherent differences between the DM population with and without HT which could contribute to differing rates of CVD-related healthcare utilization (ie, confounding factors). There are also missing data on conventional risk factors for poor CV outcomes (eg, alcohol use) that were not available in MEPS and thus cannot be accounted for. However, we considered the most relevant covariates available and employed a covariate balancing approach to mitigate variances in baseline event risk between the 2 groups in an attempt to isolate the potential effect of HT. It is essential to acknowledge that national estimates derived from weighted data may exhibit reduced reliability with smaller sample sizes, which should be taken into consideration when interpreting national utilization estimates among patients with HT. Because of the coding schemes employed in the MEPS dataset, we cannot accurately differentiate between type 1 and type 2 DM diagnoses, thus we cannot measure the effect of HT on care utilization in the separate DM populations (although we expect the large majority had type 2 DM in this study). Also, due to its nature as a national survey, MEPS data on medical diagnoses and prescription use rely on participant reporting, which may introduce certain biases, including recall bias and response bias. Renal disease, which is not categorized as a priority condition in MEPS, was almost certainly underreported, both due to the nature of the survey and that awareness of renal disease among patients is notoriously low [31]. Finally, we were unable to ascertain the degree of HT control due to the lack of thyroid function tests available in the MEPS database. Thus, we cannot differentiate between those with DM and HT who are appropriately treated and those who are undertreated or overtreated (which are associated with increased CVD risk). Data on thyroid function testing with healthcare utilization estimates would be additive in future work.

In conclusion, in this study, we found that treated HT is associated with excess CVD-related care utilization in US adults with DM even after covariate balancing. The presence of DM could pose challenges for the management of HT, potentially leading to both over- and undertreatment of HT, which could contribute to increased CVD risk in individuals with DM. Areas of future research include investigating whether treated HT correlates with biomarkers of CVD risk in DM, such as hemoglobin A1c, cholesterol levels, and renal function.

## Data Availability

Some or all datasets generated during and/or analyzed during the current study are not publicly available but are available from the corresponding author on reasonable request.

## Abbreviations

CAD: coronary artery disease
CVD: cardiovascular disease
DM: diabetes mellitus
HT: hypothyroidism
ICD: International Classification of Disease
MEPS: Medical Expenditure Panel Survey
RR: relative risk
SGLT-2: sodium-glucose cotransporter type 2
TIA: transient ischemic attack
